# Supplementary material for: Cytokine-Laden Extracellular Vesicles Predict Patient Prognosis after Cerebrovascular Accident
Source: Int J Mol Sci. 2021 Jul 22;22(15):7847. doi: 10.3390/ijms22157847 (PMC8345931; doi:10.3390/ijms22157847)
Supplement: Supplementary file 1 [file ijms-22-07847-s001.zip › Supp Table S2 new.pdf]

Glasgow Coma Scale (GCS)

Component	Response	Score
Eye Opening	Spontaneous	4
	To speech	3
	To pain	2
	None	1
Best Verbal Response	Oriented	5
	Confused	4
	Inappropriate	3
	Incomprehensible	2
	None	1
Best Motor Response	Obeying	6
	Localizes	5
	Withdraws	4
	Flexion	3
	Extension	2
	None	1

Glasgow Outcome Scale-Extended (GOSE)

GOSE 8-point scale	Domain	Criteria
1 = Dead		Dead
2 = Vegetative state	Consciousness	
3 = Lower severe disability	Function in Home	Unable to look after themselves for 8 h
4 = Upper severe disability	Function in Home<br>Function Outside the Home	Unable to look after themselves for 24 h OR<br>Unable to shop OR<br>Unable to travel
5 = Lower moderate disability	Work/Study<br>Social and Leisure Activities<br>Family and Friendships	Unable to work/study OR<br>Unable to participate OR<br>Constant problems
6 = Upper moderate disability	Work<br>Social and Leisure Activities<br>Family and Friendships	Reduced work capacity OR<br>Participate much less OR<br>Frequent problems
7 = Lower good recovery	Social and Leisure Activities<br>Family and Friendships<br>Symptoms	Participate a bit less OR<br>Occasional problems OR<br>Some symptoms affecting daily life
8 = Upper good recovery		No problems
