# Supplementary figures and images for: Cytokine-Laden Extracellular Vesicles Predict Patient Prognosis after Cerebrovascular Accident
Source: Int J Mol Sci. 2021 Jul 22;22(15):7847. doi: 10.3390/ijms22157847 (PMC8345931; doi:10.3390/ijms22157847)

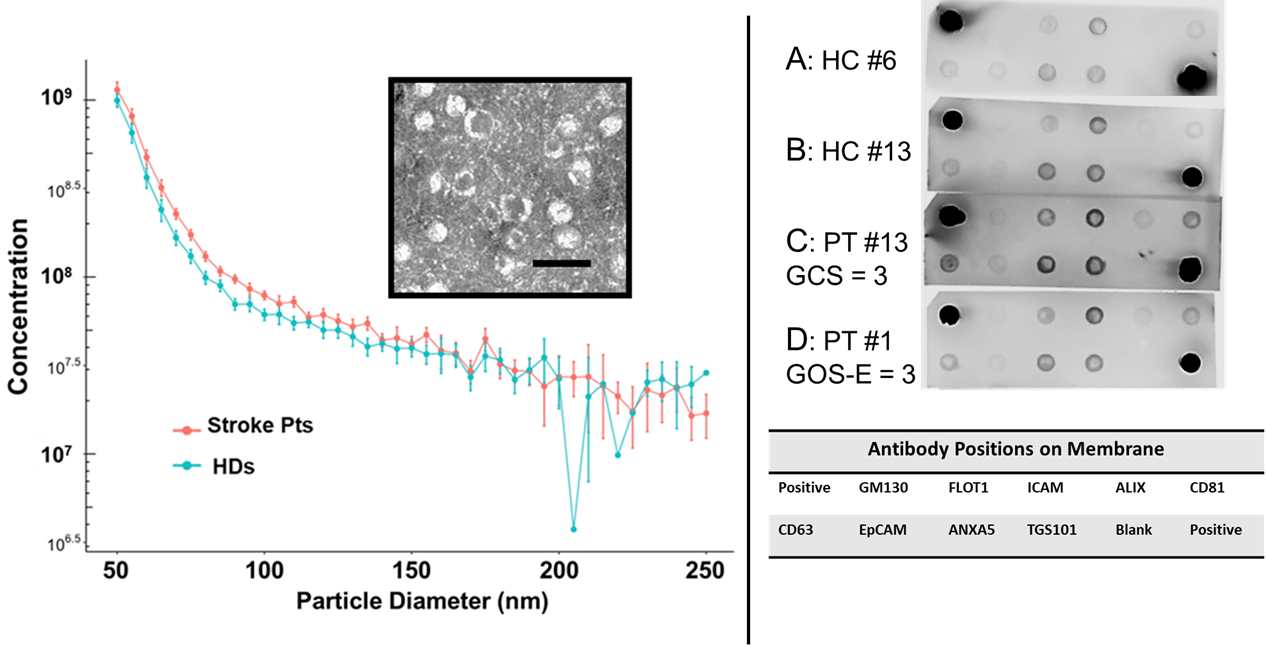

Supplement: Supplementary file 1 [file ijms-22-07847-s001.zip › Supp Figure S1.tif]

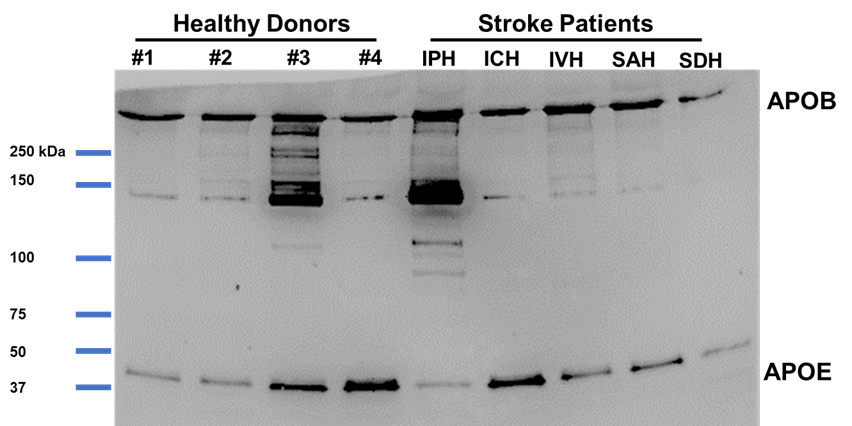

Supplement: Supplementary file 1 [file ijms-22-07847-s001.zip › Supp Figure S2.tif]

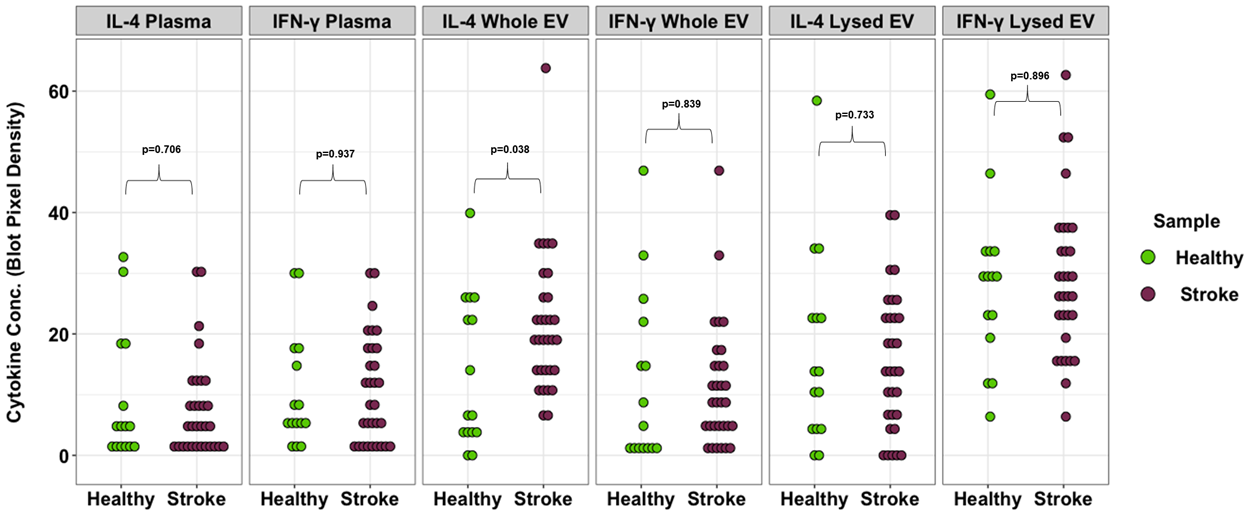

Supplement: Supplementary file 1 [file ijms-22-07847-s001.zip › Supp Figure S3.tif]

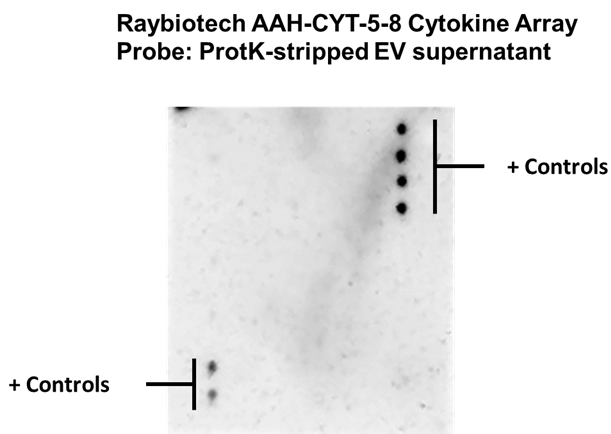

Supplement: Supplementary file 1 [file ijms-22-07847-s001.zip › Supp Figure S4.tif]

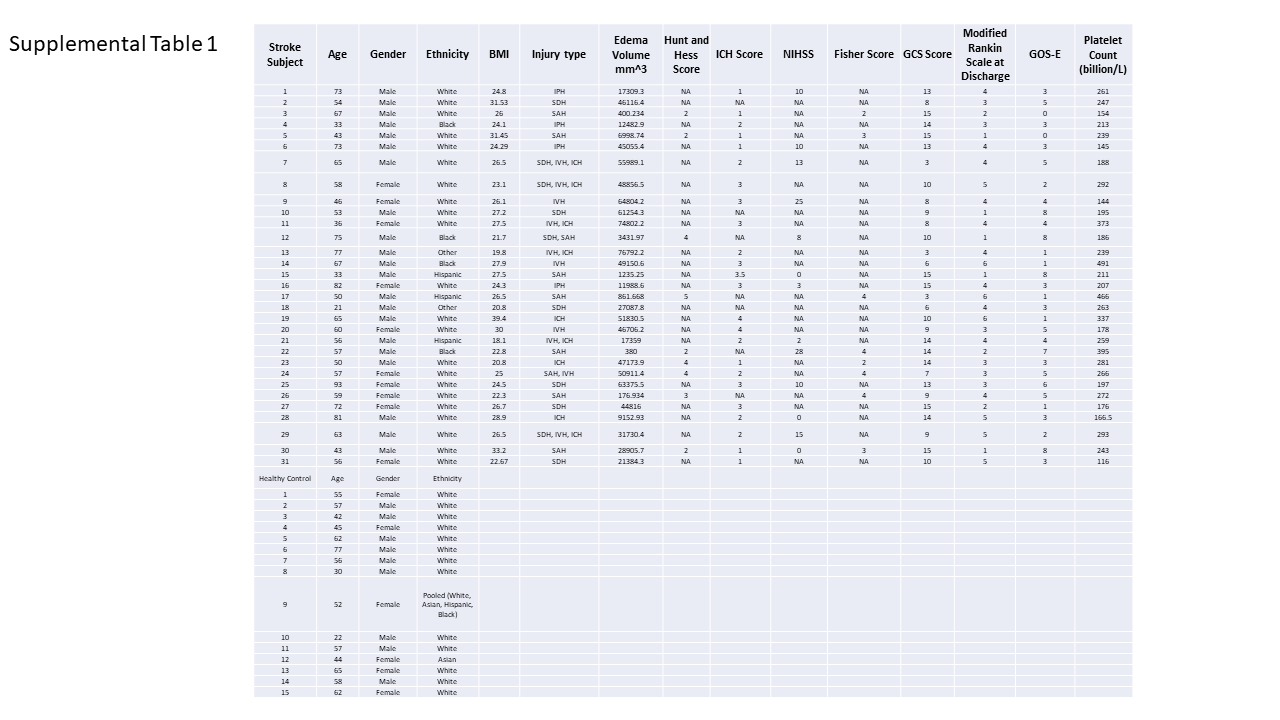

Supplement: Supplementary file 1 [file ijms-22-07847-s001.zip › Supp Table S1.tif]

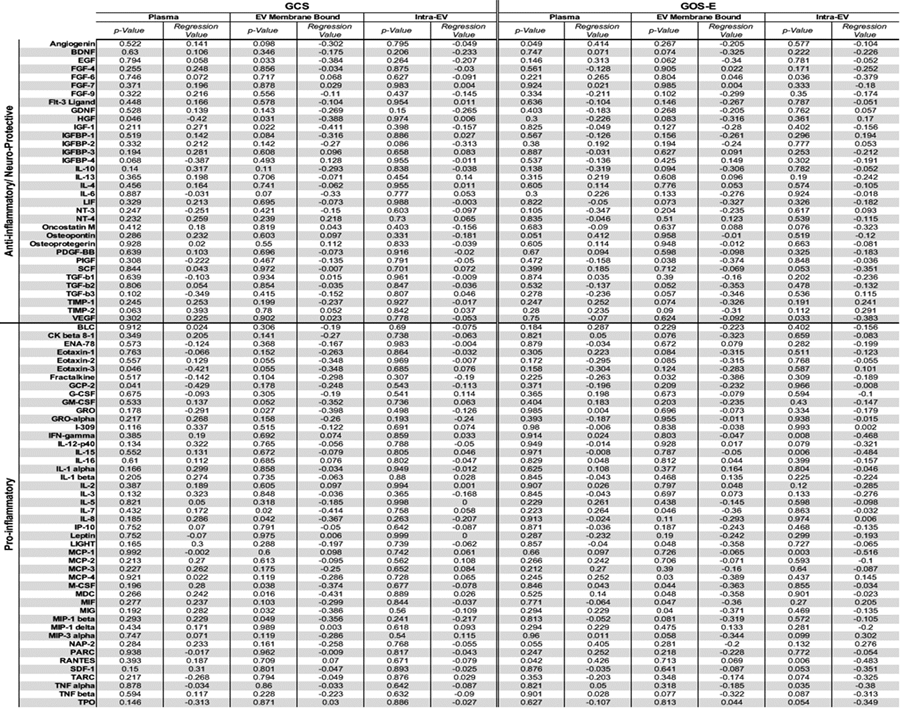

Supplement: Supplementary file 1 [file ijms-22-07847-s001.zip › Supp Table S3.tif]
